# Comparison of the prognostic values of three calculation methods for echocardiographic relative wall thickness in acute decompensated heart failure

**DOI:** 10.1186/s12947-019-0179-6

**Published:** 2019-12-03

**Authors:** Satoshi Yamaguchi, Michio Shimabukuro, Masami Abe, Tomohiro Arakaki, Osamu Arasaki, Shinichiro Ueda

**Affiliations:** 1 Department of Cardiology, Tomishiro Central Hospital, 25 Ueta, Okinawa, 901-0243 Japan; 2 Department of Clinical Pharmacology and Therapeutics, Graduate School of Medicine, University of the Ryukyus, 205 Uehara, Nishihara-cho, Okinawa, 901-0215 Japan; 3 Department of Diabetes, Endocrinology, and Metabolism, School of Medicine, Fukushima Medical University, 1 Hikarigaoka, Fukushima, 960-1295 Japan

**Keywords:** Concentric left ventricular structure, Relative wall thickness, Acute decompensated heart failure, Transthoracic echocardiography, Prognosis

## Abstract

**Purpose:**

Left ventricular (LV) wall thickness can be measured at the posterior wall (PW) and the intraventricular septum (IVS) in a parasternal long axis view by transthoracic echocardiography. Thus, there are three methods to calculate relative wall thickness as follows: RWT_PW_ = 2 × PWth/LVDd; RWT_IVS + PW_ = (IVSth + PWth) /LVDd; and RWT_IVS_ = 2 × IVSth/LVDd (IVSth = interventricular septum thickness; LVDd = LV internal dimension at end--diastole; PWth = posterior wall thickness). The aim was to compare the prognostic values of these RWTs in patients with acute decompensated heart failure (ADHF).

**Method:**

This was a single-center, retrospective, observational study at a Japanese community hospital. A total of 389 hospitalized ADHF patients were divided into two groups based on the three median RWT values. The primary outcome was all-cause death. Survival analysis was performed, and Cox proportional hazard models unadjusted and adjusted by Get With The Guideline score were used.

**Results:**

High-RWT_PW_ had poor survival (log-rank, *P* = 0.009) and was a significant risk (unadjusted HR (95%CI), 1.72 (1.14–2.61), *P* = 0.01; adjusted HR, 1.95 (1.28–2.98), *P* = 0.02). High-RWT_IVS + PW_ was not associated with poor survival on survival analysis or the unadjusted Cox model. Only the adjusted Cox model showed that High-RWT_IVS + PW_ was associated with a significant risk of the primary outcome (unadjusted HR (95%CI), 1.45 (0.96–2.17), *P* = 0.07; adjusted HR, 1.53 (1.01–2.32), *P* = 0.045). High-RWT_IVS_ did not have significant prognostic value.

**Conclusions:**

When calculating RWT, RWT_PW_ should be recommended for evaluating the mortality risk in ADHF.

## Introduction

A concentric left ventricular (LV) structure is the result of remodeling that occurs with LV wall thickening relative to the LV cavity to compensate for pressure overload [1, 2]. A concentric LV structure is a risk factor for cardiovascular events in hypertensive patients [3, 4]. Furthermore, we previously reported that a concentric LV structure evaluated by transthoracic echocardiography (TTE) was associated with poor survival in patients with acute decompensated heart failure (ADHF) [5].

Relative wall thickness (RWT) is an index of LV concentricity. RWT is the ratio of LV wall thickness to the LV internal dimension at end diastole (LVDd) [6]. LV wall thickness, which can be measured in a parasternal long-axis view by TTE, is represented by the Interventricular septum wall thickness (IVSth) and the posterior wall thickness (PWth) [6]. Therefore, there are three methods to calculate the RWT: RWT_PW_ = 2 × PWth/LVDd; RWT_IVS + PW_ = (IVSth + PWth) LVDd; and RWT_IVS_ = 2 × IVSth/LVDd. The American Society of Echocardiography (ASE) recommends RWT_PW_ for calculating RWT [6]. However, some studies found that RWT_IVS + PW_ had clinical significance [7, 8]. The difference in clinical significance among the three methods of measuring RWT is unclear.

To compare the clinical significance of RWT_PW_, RWT_IVS + PW_, and RWT_IVS_, the prognostic values of the RWTs were examined and compared in patients with ADHF.

## Materials and methods

### Participants

**Fig. 1:**
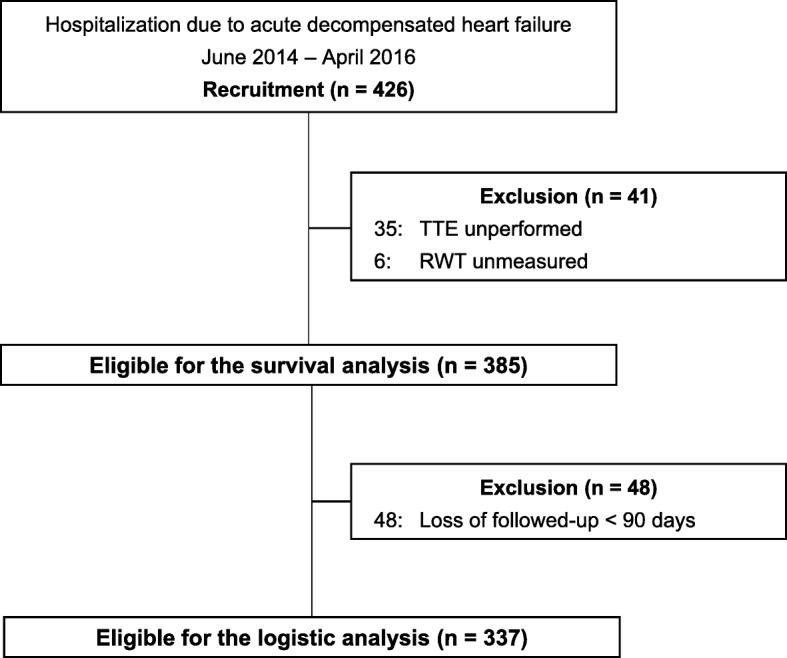
Flowchart of enrollment. RWT, relative wall thickness; TTE, transthoracic echocardiography

This was a single-center, retrospective, observational study conducted at a Japanese community hospital. In total, 426 consecutive patients admitted due to ADHF through the clinic or emergency room were recruited between June 2014 and April 2016 and followed-up from June 2014 to September 2016. A total of 41 patients were excluded for any of the following reasons: no TTE on admission (*n* = 35); and RWT not measured (*n* = 6). Finally, 385 patients were eligible for the analysis (Fig. 1). We previously documented the enrolled patients in detail [5].

The present study followed the tenets of the Declaration of Helsinki and the Ethical Guidelines for Medical and Health Research Involving Human Subjects proposed by the Ministry of Health and Welfare in Japan. The institutional ethics committee at Tomishiro Central Hospital approved the present study and waived informed consent because of the observational nature of the study.

### Transthoracic echocardiography

Comprehensive TTE (Vivid 7 ultrasound system, GE Vingmed Ultrasound, Horten, Norway) was performed during hospital admission by four medical technicians who had at least 5 years of experience performing TTE. Their measurements followed established and standardized methods recommended by the ASE and the European Society of Cardiology. At least two attending cardiologists certified by the Japanese Circulation Society and an experienced sonographer reviewed the echocardiography reports immediately after comprehensive TTE. LV geometry, including PWth, IVSth, and LVDd, was measured in M-mode in a parasternal long-axis view [6]. All measurements were performed from the leading edge to the leading edge [6]. RWTs were calculated by the three measurement methods and defined as follows: RWT_PW_ = 2 × PWth/LVDd; RWT_IVS + PW_ = (IVSth + PWth)/LVDd; and RWT_IVS_ = 2 × IVSth/LVDd. The patients were divided into two groups based on the median RWT_PW_ (low- and high-RWT_PW_), median RWT_IVS + PW_ (low- and high-RWT_IVS + PW_), or median RWT_IVS_ (low- and high-RWT_IVS_).

Left ventricular ejection fraction (LVEF) was assessed using the biplane Simpson’s method [6]. Heart failure with preserved ejection fraction (HFpEF) was defined as an ejection fraction ≥50% [9]. LV mass was computed by the Cube formula [6]. LV end-diastolic volume (LVEDVI) was estimated by the Teichholz equation [10]. Peak transmitral early diastolic wave (E wave) velocity, atrial contraction wave (A wave) velocity, and deceleration time (DCT) were measured by the pulse wave Doppler signals of the mitral inflow in the apical four-chamber view [11]. Valvular diseases were evaluated using a semiquantitative 4-grade scale (none, mild, moderate, and severe**)** [12].

### Data collection

Cardiologists followed the patients at Tomishiro Central Hospital Clinic every 1–3 months after hospital discharge. Medical clerks confirmed the patients’ condition if the patients canceled the appointment.

Patients’ medical charts were reviewed to collect their demographic characteristics and clinical data, including medications, laboratory tests, and hemodynamic data on hospital admission. The primary outcome was all-cause death. Death was confirmed by the medical chart, telephone call with a patient’s family, or obituary in local newspapers.

### Statistical analysis

Continuous variables with normal and skewed distributions are presented as means (SD) and medians [25th, 75th percentiles], respectively. Categorical variables are presented as numbers with a percentage.

In two-group comparisons, Student’s *t*-test and the Mann-Whitney U test were used to compare normally distributed and non-normally distributed continuous variables, respectively. Fisher’s exact test was used for categorical variables.

### Survival analysis

During follow-up (235 [92, 425] days), 95/385 (25%) patients died. Survival analysis for all-cause death was performed. Kaplan-Meier curves were stratified by RWT_PW_, RWT_IVS + PW_, and RWT_IVS_. The log-rank test was used to compare survival curves. High-RWT_PW_, high-RWT_IVS + PW_, and high-RWT_IVS_ were examined by univariate Cox proportional hazard models and a Cox proportional hazard model adjusted by the Get With The Guideline score (GWTG) [13, 14], an established risk score for mortality in patients with acute heart failure, to obtain hazard ratios (HRs) and 95% confidence intervals (95% CIs).

### Logistic regression model for 90-day mortality

A total of 48 patients who were lost to follow-up were excluded to evaluate the risk of 90-day mortality. Logistic regression models were used to obtain the odds ratios (ORs) of 90-day mortality and 95% CIs. High-RWT_PW_, high-RWT_IVS + PW_, and high-RWT_IVS_ were examined in univariate logistic regression models and a logistic regression model adjusted by GWTG.

### Receiver operating curves for 90-day mortality

Receiver operating curves for 90-day mortality were drawn using RWT_PW_, RWT_IVS + PW_, and RWT_IVS_ to obtain c-statistics, and the best RWT cut-off values were determined by the maximum Youden index [15].

### Sensitivity analysis of the survival analysis by stratified RWTs by the best cut-off

To confirm the consistency of the survival analysis, the participants were divided based on the best RWT cut-off value derived from the Youden index.

Survival analysis was performed to compare low and high-RWTs. High-RWT_PW_, high-RWT_IVS + PW_, and high-RWT_IVS_ were also examined with univariate and adjusted proportional Cox hazard models.

### Relationships between RWTs and clinical characteristics

Spearman’s correlation coefficient (ρ) was used to identify significant associations between RWTs and clinical characteristics: age, the natural logarithm of brain natriuretic peptide (logBNP), LVEF, LVEDV, and systolic blood pressure (SBP).

### Reliability of measurement of PWth and IVSth

The reliabilities of the TTE measurements of PWth, IVSth, and LVDd were examined in 25 patients whose TTE image quality was good, and all of the patients underwent TTE performed by the same one of four medical technicians. The medical technician and two other examiners re-measured PWth and IVSth in the TTE image stored in the local server on hospital admission, using an off-line image analysis system (Nahri Aqua, Mehergen Group, Fukuoka, Japan). Comparing every two examiners’ measurements, Bland-Altman plots were used to assess the agreement between the measurement by the same examiner and different examiners [16]. The inter-class coefficient (ICC) was computed to assess agreement [17].

The reliabilities of RWT_PW_, RWT_IVS + PW_, and RWT_IVS_ were also examined. RWTs were computed using PWth, IVSth, and LVDd measured by three examiners. Bland-Altman plots were drawn, and the ICC and *P* values were calculated.

### Software

The statistical software used was R 3.4.3 (R Foundation for Statistical Computing, Vienna, Australia). All reported *P* values are two-tailed, and a *P* value < 0.05 was considered significant.

## Results

### Participants

The participants’ median age was 81 years, and there were 181/385 (47%) men in the overall population. Comparing low- and high-RWT_PW_, high-RWT_PW_ had more elderly patients and more females, whereas in comparisons between low- and high-RWT_IVS + PW_ and between low- and high-RWT_IVS_, there were no significant differences in baseline characteristics (Table 1).

**Table 1:** Demographic data and echocardiographic parameters

	Overall	RWT_PW_^b^			RWT_IVS + PW_^b^			RWT_IVS_^b^		
		Low	High	*P* value	Low	High	*P* value	Low	High	*P* value
	*n* = 385	*n* = 193	*n* = 192		*n* = 193	*n* = 192		*n* = 193	*n* = 192	
Age, y	81 [70, 88]	80 [68, 87]	83 [73, 89]	0.021	80 [69, 87]	83 [73, 89]	0.067	80 [69, 87]	83 [73, 88]	0.082
Mele, n (%)	181/385 (47)	104/193 (54)	77/192 (40)	0.008	99/193 (51)	82/192 (43)	0.1	98/193 (51)	83/192 (43)	0.15
Height, cm	154 ± 10	156 ± 9.7	153 ± 9.9	0.002	155 ± 10	153 ± 10	0.048	155 ± 10	153 ± 10	0.15
Body weight., kg	60 ± 16	60 ± 15	60 ± 17	0.95	61 ± 15	59 ± 17	0.45	60 ± 16	59 ± 16	0.59
Body mass index, kg/m^2^	22.8 ± 4.6	22.6 ± 4.5	23.0 ± 4.8	0.42	22.8 ± 4.6	22.8 ± 4.7	0.99	22.9 ± 4.7	22.8 ± 4.6	0.87
Body surface area, m^2^	1.51 ± 0.22	1.53 ± 0.22	1.49 ± 0.23	0.11	1.53 ± 0.22	1.50 ± 0.23	0.2	1.52 ± 0.22	1.50 ± 0.22	0.3
Get With The Guideline score	38 ± 7	38 ± 6	38 ± 8	0.99	38 ± 7	38 ± 8	0.55	38 ± 7	38 ± 8	0.86
Hospital stay, days	13 [8, 20]	13 [8, 21]	12 [8, 19]	0.55	13 [8, 21]	12 [8, 19]	0.35	13 [8, 20]	12 [8, 19]	0.76
Past medical history, n (%)										
Hypertension	187/385 (49)	92/193 (48)	95/192 (50)	0.76	92/193 (48)	95/192 (50)	0.76	93/193 (48)	94/192 (49)	0.92
Diabetes mellitus	132/385 (34)	68/193 (35)	64/192 (33)	0.75	75/193 (39)	57/192 (30)	0.068	80/193 (42)	52/192 (27)	0.004
Chronic obstructive pulmonary disease	18/385 (4.7)	7/193 (3.6)	11/192 (5.7)	0.35	7/193 (3.6)	11/192 (5.7)	0.35	8/193 (4.1)	10/192 (5.2)	0.64
Old myocardial infarction	62/385 (16)	37/193 (19)	25/192 (13)	0.13	35/193 (18)	27/192 (14)	0.33	34/193 (18)	28/192 (15)	0.49
Echocardiographic parameters										
RWT_PW_^a^	0.36 ± 0.12	0.28 ± 0.05	0.45 ± 0.12	< 0.001	0.28 ± 0.05	0.44 ± 0.12	< 0.001	0.30 ± 0.07	0.43 ± 0.13	< 0.001
RWT_IVS + PW_^a^	0.37 ± 0.13	0.29 ± 0.06	0.45 ± 0.12	< 0.001	0.28 ± 0.05	0.46 ± 0.12	< 0.001	0.29 ± 0.06	0.46 ± 0.12	< 0.001
RWT_IVS_^a^	0.38 ± 0.14	0.30 ± 0.09	0.46 ± 0.15	< 0.001	0.29 ± 0.06	0.48 ± 0.14	< 0.001	0.28 ± 0.06	0.48 ± 0.13	< 0.001
IVSth, mm	9.4 ± 2.4	8.5 ± 2.0	10.4 ± 2.4	< 0.001	8.1 ± 1.8	10.7 ± 2.2	< 0.001	7.9 ± 1.5	11.0 ± 2.1	< 0.001
PWth, mm	9.0 ± 2.1	7.8 ± 1.3	10.3 ± 2.0	< 0.001	8.1 ± 1.4	10 ± 2.2	< 0.001	8.3 ± 1.6	9.8 ± 2.3	< 0.001
LVDd, mm	52 ± 9.7	57.1 ± 8.8	46.8 ± 7.8	< 0.001	57.7 ± 8.2	46.2 ± 7.6	< 0.001	57 ± 8.8	46.8 ± 7.8	< 0.001
LVEF, (%)	47 ± 17	41 ± 16	51 ± 16	< 0.001	39 ± 16	52 ± 16	< 0.001	40 ± 16	51 ± 16	< 0.001
HFpEF (LVEF ≥ 50%), n (%)	157/383 (41)	56/193 (29)	101/190 (53)	< 0.001	47/193 (24)	110/190 (58)	< 0.001	50/193 (26)	107/190 (56)	< 0.001
LVM, g	168 [131, 211]	173 [135, 211]	164 [132, 208]	0.51	174 [138, 211]	164 [123, 208]	0.18	170 [135, 207]	166 [130, 212]	0.9
LVEDV, mL	130 [92, 167]	160 [130, 194]	102 [79, 126]	< 0.001	160 [130, 194]	97 [74, 124]	< 0.001	160 [124, 194]	102 [79, 130]	< 0.001
LVM/LVEDV	1.43 ± 0.56	1.10 ± 0.23	1.75 ± 0.61	< 0.001	1.08 ± 0.20	1.77 ± 0.59	< 0.001	1.09 ± 0.21	1.76 ± 0.6	< 0.001
E wave, cm/sec	97 ± 29	97 ± 30	97 ± 28	0.94	95 ± 28	99 ± 29	0.37	98 ± 30	96 ± 28	0.58
A wave, cm/sec	76 ± 32	70 ± 29	82 ± 34	0.011	73 ± 29	79 ± 34	0.13	74 ± 30	77 ± 33	0.57
E/A	1.21 [0.84, 1.83]	1.29 [0.89, 2.04]	1.15 [0.82, 1.68]	0.18	1.21 [0.84, 1.84]	1.21 [0.86, 1.81]	0.81	1.22 [0.84, 1.83]	1.17 [0.85, 1.83]	0.6
Deceleration time, ms	150 [123, 195]	150 [121, 196]	150 [128, 192]	0.49	149 [119, 185]	150 [129, 200]	0.043	147 [118, 181]	152 [129, 201]	0.013
Aortic valve stenosis, n (%)	29/385 (7.5)	7/193 (3.6)	22/192 (12)	0.004	8/193 (4.1)	21/192 (11)	0.012	8/193 (4.1)	21/192 (11)	0.012
Aortic valve regurgitation, n (%)	24/385 (6.2)	14/193 (7.3)	10/192 (5.2)	0.53	14/193 (7.3)	10/192 (5.2)	0.53	11/193 (5.7)	13/192 (6.8)	0.68
Mitral valve regurgitation, n (%)	59/385 (15)	39/193 (20)	20/192 (10)	0.01	40/193 (21)	19/192 (9.9)	0.004	41/193 (21)	18/192 (9.4)	0.002
Laboratory data										
Blood urea nitrogen, mg/dL	24 [17, 35]	24 [17, 34]	24 [17, 36]	0.77	24 [17, 35]	24 [17, 35]	0.67	24 [17, 36]	23 [17, 35]	0.76
Creatinine, mg/dL	1.14 [0.81, 1.52]	1.15 [0.83, 1.54]	1.09 [0.79, 1.51]	0.31	1.18 [0.83, 1.56]	1.09 [0.79, 1.50]	0.22	1.17 [0.83, 1.55]	1.07 [0.79, 1.50]	0.19
Hemoglobin, g/dL	12.0 ± 2.4	12.0 ± 2.4	11.9 ± 2.4	0.84	11.9 ± 2.4	12.0 ± 2.4	0.78	11.9 ± 2.5	12 ± 2.3	0.68
Brain natriuretic peptide, pg/mL	666 [427, 1266]	737 [449, 1376]	638 [403, 1155]	0.056	765 [472, 1376]	636 [401, 1092]	0.026	683 [437, 1349]	645 [413, 1190]	0.28
Medication, n (%)										
ACE-I and/or ARB	124/285 (32)	71/193 (37)	53/192 (28)	0.069	67/193 (35)	57/192 (30)	0.34	66/193 (34)	58/192 (30)	0.47
Beta blocker	153/385 (40)	78/193 (40)	75/192 (39)	0.069	75/193 (39)	78/192 (41)	0.8	74/193 (38)	79/192 (41)	0.65
Hemodynamic data										
Systolic blood pressure, mmHg	132 ± 26	128 ± 24	135 ± 29	0.006	131 ± 24	133 ± 29	0.35	130 ± 24	134 ± 29	0.13
Diastolic blood pressure, mmHg	78 ± 21	75 ± 19	73 ± 17	0.009	74 ± 18	77 ± 21	0.17	74 ± 18	77 ± 21	0.1
Heart rate, bpm	84 ± 21	83 ± 21	84 ± 21	0.81	83 ± 19	84 ± 22	0.64	83 ± 19	83 ± 22	0.78

A wave, late mitral valve inflow velocity; *ACE-I* angiotensin converting enzyme inhibitor; *ARB* angiotensin receptor blocker; E wave, early mitral valve inflow velocity; *IVSth* interventricular septum thickness; *LVEDV* left ventricular end diastolic volume; *LVDd* left ventricular internal dimension at end-diastole; *LVEF* left ventricular ejection fraction; *LVM* left ventricular mass; *PWth* posterior wall thickness; *RWT* relative wall thickness

^a^RWT was the ratio of left ventricular wall thickness to LVDd. Left ventricular wall thickness was measured at interventricular septum as IVSth and posterior wall as PWth. Three measurement methods to compute RWT were as follows; RWT_PW_ = 2 × PWth/LVDd, RWT_IVS + PW_ = (PWth + IVSth)/LVDd, and RWT_IVS_ = 2 × IVSth/LVDd

^b^The patients were divided into two groups based on the median of RWT_PW_, RWT_IVS + PW_, and RWT_IVS_

### Transthoracic echocardiography

The mean RWT_PW_, RWT_IVS + PW_, and RWT_IVS_ values in the overall population were 0.36 ± 0.12, 0.37 ± 0.13, and 0.38 ± 0.14, respectively.

On comparing the three RWTs (low- vs. high- RWT_PW_, RWT_IVS + PW_, RWT_IVS_), high-RWTs had thicker IVSth and PWth, smaller LVDd, greater LVEF, smaller LV end-diastolic volume, high LVM/LVEDV, and less severe mitral regurgitation than low-RWTs (Table 1).

### Survival analysis

During follow-up (235 [92, 425] days), 95/385 (25%) patients died in the overall population.

**Fig. 2:**
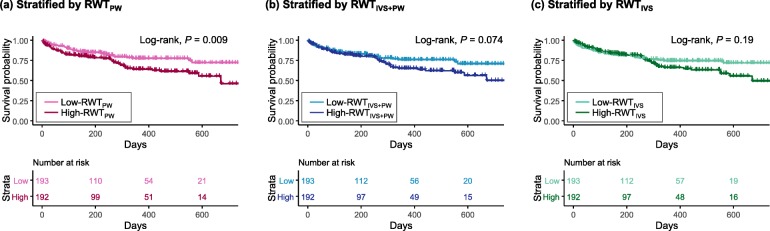
Kaplan-Meier Curves for all-cause death stratified by the RWTs. RWT, relative wall thickness. RWT_PW_ = 2 × PWth/LVDd, RWT_IVS + PW_ = (IVSth + PWth)/LVDd, and RWT_IVS_ = 2 × IVSth/LVDd. The patients were divided into two groups based on the median RWTs

Comparing low- and high-RWT_PW_, there was a significant difference in the incidence of all-cause death (low 36/193 (19%) vs. high-RWT_PW_ 59/192 (31%), *P* = 0.007). Kaplan-Meier curves showed that high-RWT_PW_ had worse survival than low-RWT_PW_ (*P* for log-rank test = 0.009; Fig. 2a).

Comparing low- and high-RWT_IVS + PW_, there was no significant difference in all-cause death (low 40/193 (21%) vs. high-RWT_PW_ 55/192 (29%), *P* = 0.077) or survival (*P* for log-rank test = 0.074; Fig. 2b).

In a comparison between low- and high-RWT_IVS_, there was no significant difference in all-cause death (low 42/193 (22%) vs. high-RWT_IVS_ 53/192 (28%), *P* incidence = 0.2) or survival (*P* for log-rank test = 0.19; Fig. 2c).

### Cox proportional hazard models for all-cause death

In the unadjusted and adjusted Cox proportional hazard models, high-RWT_PW_ was a significant risk factor for all-cause death (unadjusted Cox model, HR (95% CI), 1.72 (1.41–2.61), *P* = 0.01; adjusted Cox model, 1.95 (1.28–2.98), *P* = 0.02; Table 2).

**Table 2:** Cox proportional hazard model for evaluate the risk of RWTs for all-cause mortality

Calculate method and factor	Unadjusted						Adjusted by GWTG					
	Event/cases	HR	95% CI			*P* value	Event/cases^a^	HR	95% CI			*P* value
High- to low-RWT_PW_	95/385	1.72	1.14	–	2.61	0.01	93/380	1.95	1.28	–	2.98	0.002
High- to low-RWT_IVS + PW_	95/385	1.45	0.96	–	2.17	0.075	93/380	1.53	1.01	–	2.32	0.045
High- to low-RWT_IVS_	95/385	1.31	0.87	–	1.96	0.19	93/380	1.36	0.9	–	2.06	0.14

*CI* confidence interval; *GWTG* Get With The Guideline score; *HR* hazard ratio; *RWT* relative wall thickness

^a^5 cases were removed because of GWTG missing

High-RWT_IVS + PW_ was not a significant risk factor for all-cause death in the unadjusted Cox proportional model (unadjusted Cox model, HR, 1.45 (0.96–2.17), *P* = 0.075), but it was in the adjusted Cox proportional hazard model (adjusted Cox model, 1.53 (1.01–2.32), *P* = 0.045; Table 2).

High-RWT_IVS_ was not a significant factor in either the unadjusted or the adjusted Cox proportional hazard model (Table 2).

### Logistic regression models for 90-day mortality

The OR of high- to low-RWT_PW_ was significant (univariate, OR, 2.19, 95%CI, 1.15–2.19, *P* = 0.017; adjusted, OR, 2.26, 95%CI, 1.16–4.4, P = 0.017) on univariate analysis and the adjusted logistic regression model (Table 3). In contrast, the OR of neither high to low-RWT_IVS + PW_ nor RWT_IVS_ was significant on univariate analysis or the adjusted logistic regression models.

**Table 3:** Logistic models for evaluating the risk of 90 days mortality

Calculate method and factor	Unadjusted						Adjusted by GWTG					
	Event/cases	OR	95% CI			*P* value	Event/cases	OR	95% CI			*P* value
High- to low-RWT_PW_	48/337	2.19	1.15	–	2.19	0.017	48/337	2.26	1.16	–	4.4	0.017
High- to low-RWT_IVS + PW_	48/337	1.26	0.68	–	1.26	0.46	48/337	1.19	0.63	–	2.25	0.6
High- to low-RWT_IVS_	48/337	0.86	0.47	–	0.86	0.64	48/337	0.8	0.42	–	1.52	0.5

*CI* confidence interval; *GWTG* Get With The Guideline score, *OR* odds ratio; *RWT* relative wall thickness

### Receiver operating curves for 90-day mortality

**Fig. 3:**
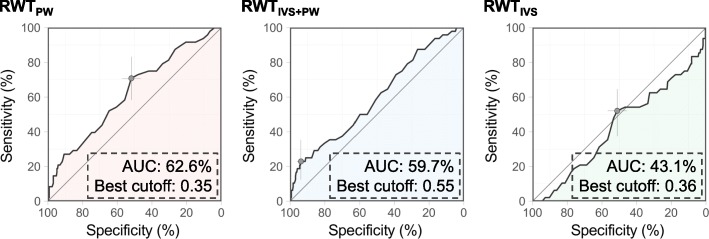
Receiver operating curves for 90-day mortality using the RWTs. AUC, area under the curve

A total of 48 (13%) patients died within 90 days from hospital admission. Figure 3 shows the receiver operating characteristic (ROC) curves for 90-day mortality using the RWTs. The c-statistic of the ROC curve using RWT_PW_ was 62.6%, and the best cut-off value was 0.35. The c-statistic of the ROC curve using RWT_IVS + PW_ was 59.7%, and the best cut-off value was 0.55. The c-statistic of the ROC curve using RWT_IVS_ was 43.1%, and the best cut-off value was 0.36.

### Sensitivity analysis of the survival analysis by stratified RWTs by the best cut-off

Additional file 1: Table S1 shows the demographic data and echocardiographic data with stratification by the best RWT cut-off. High-RWT_PW_ had worse survival than low-RWT_PW_ (*P* for log-rank test = 0.03; Additional file 2: Figure S1a). High-RWT_IVS + PW_ also had a worse prognosis than low-RWT_IVS + PW_ (*P* for log-rank test < 0.001; Additional file 2: Figure S1b). In contrast, there was no significant difference in survival between low- and high-RWT_IVS_ (*P* for log-rank test = 0.077; Additional file 2: Figure S1c).

In the unadjusted and adjusted Cox proportional hazard models, high-RWT_PW_ and high-RWT_IVS + PW_ were associated with mortality (high-RWT_PW_, unadjusted Cox model, HR (95% CI), 1.55 (1.04–2.33), *P* = 0.033; adjusted Cox model, 1.72 (1.14–2.59), *P* = 0.01; high-RWT_IVS + PW_, unadjusted Cox model, HR (95% CI), 3.88 (2.34–6.43), *P* <  0.001; adjusted Cox model, 3.42 (2.04–5.72), *P* <  0.001; Additional file 3: Table S2). High-RWT_IVS_ was not a significant risk factor in the unadjusted and adjusted Cox proportional hazard models.

### Relationship between RWTs and clinical characteristics

There were significant positive correlations between the three RWTs and age and LVEF, and negative correlations between the RWTs and LogBNP and LVEDV (Table 4). RWT_IVS + PW_ and RWTI_VS_ did not have significant correlations with systolic blood pressure, but RWT_PW_ did (ρ = 0.15, *P* = 0.004).

**Table 4:** Relationship between RWTs and clinical characteristics

	RWT_PW_		RWT_IVS + PW_		RWT_IVS_	
	ρ	*P* value	ρ	*P* value	ρ	*P* value
Age, y	0.15	0.003	0.17	0.003	0.17	0.001
LogBNP, log (pg/mL)	−0.2	< 0.001	−0.15	0.003	−0.11	0.039
LVEF, %	0.42	< 0.001	0.47	< 0.001	0.43	< 0.001
LVEDV, mL	−0.67	< 0.001	−0.74	< 0.001	− 0.69	< 0.001
Systolic blood pressure, mmHg	0.15	0.004	0.094	0.065	0.063	0.22

*LogBNP* logarithmed brain natriuretic peptide; *LVEDV* left ventricular end-diastolic volume; *LVEF* left ventricular ejection fraction; ρ, Spearman’s correlation coefficient

### Reliability of TTE measurement of PWth, IVSth, and LVDd

**Fig. 4:**
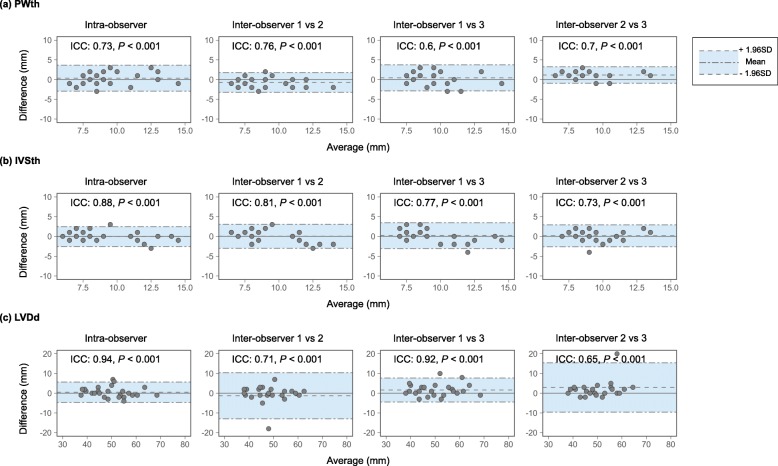
Reliability of linear measurements of PWth, IVSth, and LVDd. IVSth, interventricular septum thickness; LVDd, left ventricular internal dimension at end-diastole; PWth, posterior wall thickness; ICC, intraclass correlation coefficient

Intra-observer agreement of TTE measurement of PWth was significant (ICC = 0.73, *P* <  0.001; Fig. 4a). Inter-observer agreements of TTE measurement of PWth were also significant (observer 1 vs. 2, ICC = 0.76, *P* <  0.001; observer 1 vs. 3, ICC = 0.6, *P* <  0.001; observer 2 vs. 3, ICC = 0.7, *P* <  0.001; Fig. 3a). There were no systematic biases in the intra- and inter-observer agreements in PWth measurement (Fig. 4a).

Intra-observer agreement of TTE measurement of IVSth was significant (ICC = 0.88, *P* <  0.001; Fig. 4b). Inter-observer agreements of TTE measurement of IVSth were also significant (observer 1 vs. 2, ICC = 0.81, *P* <  0.001; observer 1 vs. 3, ICC = 0.77, *P* <  0.001; observer 2 vs. 3, ICC = 0.73, *P* <  0.001; Fig. 4b). There were no systematic biases in the intra- and inter-observer agreements in IVSth measurement (Fig. 4b).

Intra-observer agreement of TTE measurement of LVDd was significant (ICC = 0.94, *P* <  0.001; Fig. 4c). Inter-observer agreements of TTE measurement of LVDd were also significant (observer 1 vs. 2, ICC = 0.71, *P* <  0.001; observer 1 vs. 3, ICC = 0.92, *P* <  0.001; observer 2 vs. 3, ICC = 0.65, *P* <  0.001; Fig. 4c). There were no systematic biases in the intra- and inter-observer agreements in LVDd measurement (Fig. 4c).

### Reliability of RWTs obtained from TTE measurement

**Fig. 5:**
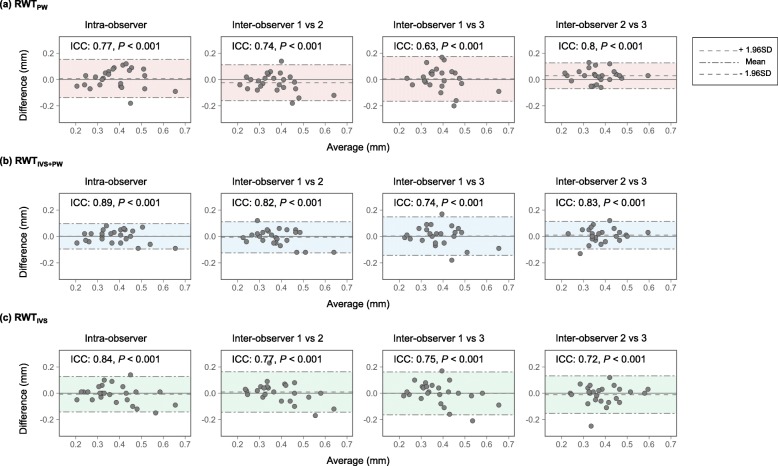
Reliability of RWTs. RWT, relative wall thickness. RWT_PW_ = 2 × PWth/LVDd, RWT_IVS + PW_ = (IVSth + PWth)/LVDd, and RWT_IVS_ = 2 × IVSth/LVDd in which PWth = posterior wall thickness, IVSth = interventricular septum thickness, and LVDd = left ventricular internal dimension at end-diastole

Intra-observer agreement of RWT_PW_ was significant (ICC = 0.77, *P* <  0.001; Fig. 5a). Inter-observer agreements of RWT_PW_ were significant (observer 1 vs. 2, ICC = 0.74, *P* <  0.001; observer 1 vs. 3, ICC = 0.63, *P* <  0.001; observer 2 vs. 3, ICC = 0.8, *P* <  0.001). There were no systematic biases in the intra- and inter-observer agreements in RWT_PW_.

Intra-observer agreement of RWT_IVS + PW_ was significant (ICC = 0.89, *P* <  0.001; Fig. 5b). Inter-observer agreements of RWT_PW_ were also significant (observer 1 vs. 2, ICC = 0.82, *P* <  0.001; observer 1 vs. 3, ICC = 0.74, *P* <  0.001; observer 2 vs. 3, ICC = 0.83, *P* <  0.001). There were no systematic biases in the intra- and inter-observer agreements in RWT_IVS + PW_.

Intra-observer agreement of RWT_IVS_ was significant (ICC = 0.84, *P* <  0.001; Fig. 5c). Inter-observer agreements of RWT_IVS_ were also significant (observer 1 vs. 2, ICC = 0.77, *P* <  0.001; observer 1 vs. 3, ICC = 0.75, *P* <  0.001; observer 2 vs. 3, ICC = 0.72, *P* <  0.001). There were no systematic biases in the intra- and inter-observer agreements in RWT_IVS_.

## Discussion

To the best of our knowledge, this is the first study to show the difference in the clinical significance of the three RWTs. The present study demonstrated that, compared to RWT_IVS + PW_ and RWT_IVS_, RWT_PW_ is the best to stratify the risk for all-cause death in ADHF patients. This may be consistently supported by three findings. First, high-RWT_PW_ had a significantly worse prognosis than low-RWT_PW_. In contrast, on survival analysis, there was no significant difference between high- and low-RWT_IVS + PW_ or RWT_IVS_. Second, in the logistic regression model for 90-day mortality, only high-RWT_PW_ was significant among the three RWTs (Table 3). Third, ROC for 90-day all-cause death using RWT_PW_ had the highest c-statistic among the three ROCs.

### Explanations of the differences in the prognostic values among the three RWTs

High-RWT_PW_ was associated with a poor prognosis on survival analysis and Cox proportional hazard models (Fig. 2a; Table 2). High-RWT_IVS + PW_ was not associated with poor survival on survival analysis (Fig. 1b), whereas high-RWT_IVS + PW_ was a significant risk only in the Cox proportional hazard model adjusted by GWTG, not in the unadjusted model (Table 2). High-RWT_IVS_ did not show worse survival than low-RWT_IVS_ (Fig. 1c; Table 2). The equations of RWT_PW_ and RWT_IVS + PW_ contain PWth. PWth or the ratio of PWth to LVDd, therefore, may represent the LV remodeling related to a worse prognosis better than IVSth or IVSth to LVDd in patients with ADHF. Patients with high-RWT_PW_ had higher systolic blood pressure than those with low-RWT_PW_ (Table 1), while there was no such difference either between low- and high-RWT_IVS + PW_ or between low- and high-RWT_IVS_. RWT_PW_ had a positive correlation with systolic blood pressure (Table 4), while either RWT_IVS + PW_ or RWT_IVS_ did not. This may suggest that thickening of PWth, rather than IVSth, is likely to counterbalance pressure overload and may lead to LV diastolic dysfunction leading to a poor prognosis. A higher A wave in high RWT_pw_ patients than in low RWT_pw_ patients may support this assumption (Table 1).

In terms of methodological validity, there were no differences in inter- and intra-observer agreements for each RWT. Given that fairly good reproducibility was observed in all measurements, differences in prognostic values among the three RWTs may not result from technical aspects of TTE.

Paradoxically, high-RWT_PW_ patients had lower BNP than low-RWT_PW_ patients (Table 1). High-RWT_PW_ included 101 (53%) patients with HFpEF. Generally, BNP increases modestly in HFpEF [18]. Furthermore, the prognostic value of BNP has not been confirmed in patients with HFpEF [19]. High RWT_PW_ might be of clinically utility, especially, in patients with HFpEF.

## Limitations

The present study had several limitations. The present study did not have pressure data such as LV end-diastolic pressure or pulmonary artery wedge pressure. LV wall thickness was not evaluated by other modalities, such as magnetic resonance imaging or computed tomography. Patients having valvular diseases with various etiologies were included, which might affect the prognostic value of RWTs.

In conclusion, high-RWT_PW_ had a higher systolic pressure and A wave than low-RWT_PW_. This finding was not observed in the comparison between low- and high-RWT_IVS + PW_ or between low-and high-RWT_IVS_. PWth may represent pressure overload better than IVSth. When calculating RWT, RWT_PW_ should be recommended for evaluating the mortality risk in ADHF.

## Supplementary information

**Additional file 1: Table S1.** Demographic data and echocardiographic parameters.

**Additional file 2: Figure S1.** Kaplan-Meier Curves for all-cause mortality stratified by the stratified RWTs by the best cut-off.

**Additional file 3: Table S2.** Cox proportional hazard model for evaluate the risk of high RWTs for all-cause mortality.

## Data Availability

Not available. We are not allowed the any study data to share by the ethical committee.
