# Supplementary material for: Comparison of the prognostic values of three calculation methods for echocardiographic relative wall thickness in acute decompensated heart failure
Source: Cardiovasc Ultrasound. 2019 Dec 3;17:30. doi: 10.1186/s12947-019-0179-6 (PMC6891973; doi:10.1186/s12947-019-0179-6)
Supplement: Supplementary file 1 — Additional file 1: Table S1. Demographic data and echocardiographic parameters. [file 12947_2019_179_MOESM1_ESM.docx]

**Table S1.** Demographic data and echocardiographic parameters.

|  | Overall |  | RWT_PW_‡ | | |  | RWT_IVS+PW_‡ | | |  | RWT_IVS_‡ | | |
| --- | --- | --- | --- | --- | --- | --- | --- | --- | --- | --- | --- | --- | --- |
|  |  |  | Low | High | P value |  | Low | High | P value |  | Low | High | P value |
|  | n = 385 |  | n = 207 | n = 178 |  |  | n = 356 | n = 29 |  |  | n = 202 | n = 183 |  |
| Age, y | 81 [70, 88] |  | 80 [69, 87] | 82 [72, 89] | 0.096 |  | 81 [71, 88] | 80 [69, 92] | 0.73 |  | 80 [68, 87] | 83 [73, 89] | 0.023 |
| Mele, n (%) | 181/385 (47) |  | 107/207 (52) | 74/178 (42) | 0.052 |  | 169/356 (48) | 12/29 (41) | 0.57 |  | 104/202 (52) | 77/183 (42) | 0.067 |
| Height, cm | 154 ± 10 |  | 155 ± 9.7 | 153 ± 10 | 0.014 |  | 154 ± 10 | 153 ± 11 | 0.54 |  | 155 ± 10 | 153 ± 10 | 0.036 |
| Body weight., kg | 60 ± 16 |  | 59 ± 15 | 60 ± 17 | 0.53 |  | 60 ± 16 | 57 ± 15 | 0.42 |  | 61 ± 16 | 59 ± 16 | 0.34 |
| Body mass index, kg/m^2^ | 22.8 ± 4.6 |  | 22.6 ± 4.5 | 23.1 ± 4.8 | 0.24 |  | 22.8 ± 4.7 | 22.6 ± 4.5 | 0.8 |  | 22.9 ± 4.6 | 22.7 ± 4.7 | 0.77 |
| Body surface area, m^2^ | 1.51 ± 0.22 |  | 1.52 ± 0.22 | 1.50 ± 0.23 | 0.33 |  | 1.51 ± 0.22 | 1.49 ± 0.23 | 0.57 |  | 1.53 ± 0.22 | 1.49 ± 0.23 | 0.12 |
| Get With The Guideline score | 38 ± 7 |  | 38 ± 7 | 38 ± 8 | 0.96 |  | 38 ± 7 | 40 ± 11 | 0.26 |  | 38 ± 6 | 38 ± 8 | 0.72 |
| Hospital stay, days | 13 [8, 20] |  | 13 [8, 21] | 12 [8, 19] | 0.34 |  | 13 [8, 20] | 13 [8, 31] | 0.26 |  | 13 [8, 21] | 12 [8, 19] | 0.76 |
| **Past medical history, n (%)** |  |  |  |  |  |  |  |  |  |  |  |  |  |
| Hypertension | 187/385 (49) |  | 97/207 (47) | 90/178 (50) | 0.48 |  | 172/356 (48) | 15/29 (52) | 0.85 |  | 96/202 (48) | 91/183 (50) | 0.68 |
| Diabetes mellitus | 132/385 (34) |  | 72/207 (35) | 60/178 (34) | 0.83 |  | 124/356 (35) | 8/29 (28) | 0.54 |  | 83/202 (41) | 49/183 (27) | 0.004 |
| Chronic obstructive pulmonary disease | 18/385 (4.7) |  | 7/207 (3.4) | 11/178 (6.2) | 0.23 |  | 15/356 (4.2) | 3/29 (10) | 0.15 |  | 8/202 (4) | 10/183 (5.2) | 0.63 |
| Old myocardial infarction | 62/385 (16) |  | 40/207 (19) | 22/178 (12) | 0.071 |  | 58/356 (16) | 4/29 (14) | 1 |  | 34/202 (17) | 28/183 (15) | 0.78 |
| **Echocardiographic parameters** |  |  |  |  |  |  |  |  |  |  |  |  |  |
| RWT_PW_† | 0.36 ± 0.12 |  | 0.28 ± 0.05 | 0.46 ± 0.12 | < 0.001 |  | 0.34 ± 0.08 | 0.64 ± 0.18 | < 0.001 |  | 0.30 ± 0.07 | 0.43 ± 0.13 | < 0.001 |
| RWT_IVS+PW_† | 0.37 ± 0.13 |  | 0.30 ± 0.06 | 0.46 ± 0.13 | < 0.001 |  | 0.35 ± 0.08 | 0.68 ± 0.16 | < 0.001 |  | 0.29 ± 0.06 | 0.46 ± 0.12 | < 0.001 |
| RWT_IVS_† | 0.38 ± 0.14 |  | 0.31 ± 0.09 | 0.47 ± 0.15 | < 0.001 |  | 0.35 ± 0.10 | 0.72 ± 0.16 | < 0.001 |  | 0.28 ± 0.06 | 0.49 ± 0.13 | < 0.001 |
| IVSth, mm | 9.4 ± 2.4 |  | 8.5 ± 2.0 | 10.5 ± 2.4 | < 0.001 |  | 9.1 ± 2.0 | 13.7 ± 2.7 | < 0.001 |  | 8.0 ± 1.6 | 11.0 ± 2.1 | < 0.001 |
| PWth, mm | 9.0 ± 2.1 |  | 7.9 ± 1.3 | 10.4 ± 2.4 | < 0.001 |  | 8.8 ± 1.4 | 12.3 ± 3.0 | < 0.001 |  | 8.3 ± 1.6 | 9.9 ± 2.3 | < 0.001 |
| LVDd, mm | 52 ± 9.7 |  | 56.7 ± 8.7 | 46.5 ± 7.8 | < 0.001 |  | 53.0 ± 9.1 | 39.3 ± 8.2 | < 0.001 |  | 56.9 ± 8.6 | 46.5 ± 7.9 | < 0.001 |
| Let ventricular ejection fraction, (%) | 47 ± 17 |  | 41 ± 16 | 52 ± 16 | < 0.001 |  | 45 ± 17 | 50 ± 17 | 0.16 |  | 40 ± 16 | 52 ± 16 | < 0.001 |
| EF ≥ 50%, n (%) | 157/383 (41) |  | 60/207 (29) | 97/178 (55) | < 0.001 |  | 140/356 (40) | 17/29 (59) | 0.07 |  | 53/202 (26) | 104/183 (58) | < 0.001 |
| LVM, g | 168 [131, 211] |  | 171 [135, 210] | 164 [132, 211] | 0.7 |  | 169 [132, 207] | 160 [116, 291] | 0.7 |  | 170 [135, 209] | 164 [128, 211] | 0.67 |
| LVEDV, mL | 130 [ 92, 167] |  | 154 [124, 194] | 97 [79, 124] | < 0.001 |  | 133 [97, 169] | 70 [47, 92] | < 0.001 |  | 154 [124, 194] | 97 [77, 127] | < 0.001 |
| LVM/LVEDV | 1.43 ± 0.56 |  | 1.12 ± 0.24 | 1.78 ± 0.61 | < 0.001 |  | 1.31 ± 0.33 | 2.82 ± 0.86 | < 0.001 |  | 1.10 ± 0.22 | 1.78 ± 0.60 | < 0.001 |
| E wave, cm/sec | 97 ± 29 |  | 96 ± 29 | 98 ± 28 | 0.59 |  | 97 ± 28 | 96 ± 35 | 0.84 |  | 98 ± 29 | 96 ± 28 | 0.63 |
| A wave, cm/sec | 76 ± 32 |  | 70 ± 29 | 83 ± 34 | 0.008 |  | 75 ± 30 | 90 ± 44 | 0.091 |  | 74 ± 30 | 77 ± 34 | 0.46 |
| E/A | 1.21 [0.84, 1.83] |  | 1.29 [0.89, 2.04] | 1.12 [0.80, 1.68] | 0.15 |  | 1.25 [0.87, 1.84] | 1.92 [0.72, 1.27] | 0.057 |  | 1.25 [0.85, 1.81] | 1.16 [0.84, 1.84] | 0.48 |
| Deceleration time, ms | 150 [123, 195] |  | 150 [121, 195] | 150 [129, 192] | 0.32 |  | 150 [121, 191] | 158 [140, 221] | 0.053 |  | 149 [118, 188] | 151 [129, 200] | 0.026 |
| Aortic valve stenosis, n (%) | 29/385 (7.5) |  | 8/207 (3.9) | 21/178 (12) | 0.004 |  | 25/356 (7) | 4/29 (14) | 0.26 |  | 8/202 (4) | 21/183 (12) | 0.006 |
| Aortic valve regurgitation, n (%) | 24/385 (6.2) |  | 15/207 (7.2) | 9/178 (5.2) | 0.41 |  | 23/356 (6.5) | 1/29 (3.4) | 1 |  | 11/202 (5.4) | 13/183 (7.1) | 0.53 |
| Mitral valve regurgitation, n (%) | 59/385 (15) |  | 40/207 (19) | 19/178 (11) | 0.023 |  | 57/356 (16) | 2/29 (6.9) | 0.28 |  | 42/202 (21) | 17/183 (9.3) | 0.002 |
| **Laboratory data** |  |  |  |  |  |  |  |  |  |  |  |  |  |
| Blood urea nitrogen, mg/dL | 24 [17, 35] |  | 24 [17, 34] | 24 [17, 35] | 0.95 |  | 24 [17, 35] | 27 [19, 41] | 0.52 |  | 24 [17, 36] | 23 [17, 35] | 0.62 |
| Creatinine, mg/dL | 1.14 [0.81, 1.52] |  | 1.15 [0.80, 1.53] | 1.09 [0.81, 1.50] | 0.44 |  | 1.14 [0.81, 1.52] | 1.19 [0.72, 1.65] | 0.58 |  | 1.17 [0.83, 1.58] | 1.09 [0.79, 1.48] | 0.14 |
| Hemoglobin, g/dL | 12.0 ± 2.4 |  | 12.0 ± 2.4 | 11.9 ± 2.4 | 0.79 |  | 11.9 ± 2.4 | 12.7 ± 2.1 | 0.072 |  | 11.9 ± 2.5 | 12 ± 2.4 | 0.65 |
| Brain natriuretic peptide, pg/mL | 666 [427, 1266] |  | 765 [469, 1413] | 627 [387, 1041] | 0.009 |  | 666 [432, 1271] | 685 [368, 1183] | 0.67 |  | 682 [444, 1338] | 651 [409, 1203] | 0.28 |
| **Medication, n (%)** |  |  |  |  |  |  |  |  |  |  |  |  |  |
| ACE-I and/or ARB | 124/285 (32) |  | 74/207 (36) | 50/178 (28) | 0.14 |  | 119/356 (33) | 5/29 (17) | 0.11 |  | 67/202 (33) | 57/183 (31) | 0.75 |
| Beta blocker | 153/385 (40) |  | 85/207 (41) | 68/178 (39) | 0.64 |  | 145/356 (41) | 8/29 (28) | 0.23 |  | 77/202 (38) | 76/183 (42) | 0.56 |
| **Hemodynamic data** |  |  |  |  |  |  |  |  |  |  |  |  |  |
| Systolic blood pressure, mmHg | 132 ± 26 |  | 128 ± 24 | 136 ± 28 | 0.008 |  | 132 ± 25 | 131 ± 38 | 0.93 |  | 130 ± 24 | 134 ± 29 | 0.13 |
| Diastolic blood pressure, mmHg | 78 ± 21 |  | 73 ± 18 | 78 ± 21 | 0.014 |  | 75 ± 19 | 80 ± 24 | 0.19 |  | 74 ± 18 | 77 ± 21 | 0.11 |
| Heart rate, bpm | 84 ± 21 |  | 83 ± 20 | 84 ± 21 | 0.45 |  | 83 ± 21 | 87 ± 20 | 0.31 |  | 83 ± 19 | 84 ± 22 | 0.8 |

High RWTs was defined as the best-cut off value of RWTs determined by Youden index in ROCs for 90 days mortality.

A wave, late mitral valve inflow velocity; ACE-I, angiotensin converting enzyme inhibitor; ARB, angiotensin receptor blocker; E wave, early mitral valve inflow velocity; IVSth, intraventricular septum thickness; LVEDV, left ventricular end diastolic volume; LVDd, left ventricular internal dimension at end diastole; LVM, left ventricular mass; PWth, posterior wall thickness; RWT, relative wall thickness.

† RWT was the ratio of left ventricular wall thickness to LVDd. Left ventricular wall thickness was measured at intraventricular septum as IVSth and posterior wall as PWth. Three measurement methods to compute RWT were as follows;

RWT_PW_ = 2 × PWth/LVDd, RWT_IVS+PW_ = (PWth + IVSth)/LVDd, and RWT_IVS_ = 2 × IVSth/LVDd.

‡ The patients were divided into two groups based on the median of RWT_PW_, RWT_IVS+PW_, and RWT_IVS_.
