# Supplementary material for: Comparison of the prognostic values of three calculation methods for echocardiographic relative wall thickness in acute decompensated heart failure
Source: Cardiovasc Ultrasound. 2019 Dec 3;17:30. doi: 10.1186/s12947-019-0179-6 (PMC6891973; doi:10.1186/s12947-019-0179-6)
Supplement: Supplementary file 2 — Additional file 2: Figure S1. Kaplan-Meier Curves for all-cause mortality stratified by the stratified RWTs by the best cut-off. [file 12947_2019_179_MOESM2_ESM.pptx]

## Slide 1
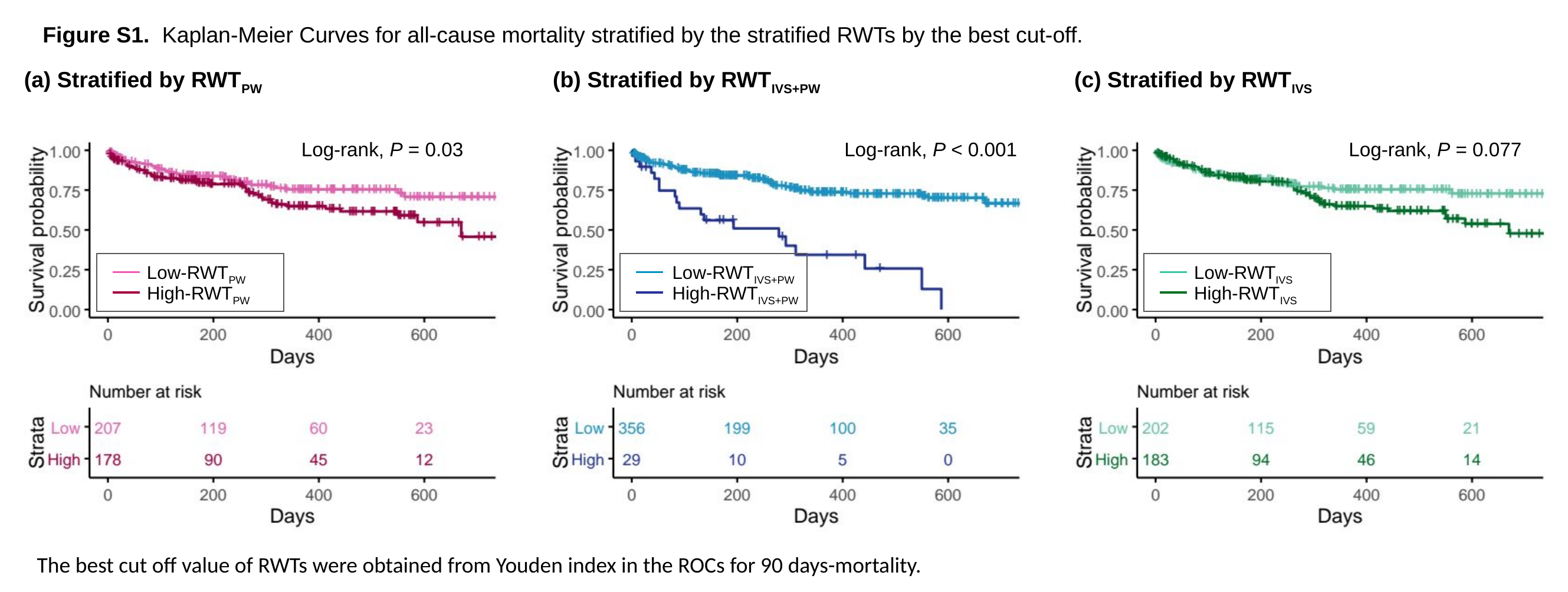

Figure S1. Kaplan-Meier Curves for all-cause mortality stratified by the stratified RWTs by the best cut-off.
(a) Stratified by RWTPW
(b) Stratified by RWTIVS+PW
(c) Stratified by RWTIVS
Log-rank, P = 0.03
Log-rank, P < 0.001
Log-rank, P = 0.077
Low-RWTPW
High-RWTPW
Low-RWTIVS
High-RWTIVS
Low-RWTIVS+PW
High-RWTIVS+PW
The best cut off value of RWTs were obtained from Youden index in the ROCs for 90 days-mortality.
