# Supplementary material for: Comparison of the prognostic values of three calculation methods for echocardiographic relative wall thickness in acute decompensated heart failure
Source: Cardiovasc Ultrasound. 2019 Dec 3;17:30. doi: 10.1186/s12947-019-0179-6 (PMC6891973; doi:10.1186/s12947-019-0179-6)
Supplement: Supplementary file 3 — Additional file 3: Table S2. Cox proportional hazard model for evaluate the risk of high RWTs for all-cause mortality. [file 12947_2019_179_MOESM3_ESM.docx]

**Table S2.** Cox proportional hazard model for evaluate the risk of high RWTs for all-cause mortality.

|  | Unadjusted | | | | | |  | Adjusted by GWTG | | | | | |
| --- | --- | --- | --- | --- | --- | --- | --- | --- | --- | --- | --- | --- | --- |
| Calculate method and factor | Event/cases | HR | 95% CI | | | P value |  | Event/cases† | HR | 95% CI | | | P value |
| High- to low-RWT_PW_ | 95/385 | 1.55 | 1.04 | - | 2.33 | 0.033 |  | 93/380 | 1.72 | 1.14 | - | 2.59 | 0.01 |
| High- to low-RWT_IVS+PW_ | 95/385 | 3.88 | 2.34 | - | 6.43 | < 0.001 |  | 93/380 | 3.42 | 2.04 | - | 5.72 | < 0.001 |
| High- to low-RWT_IVS_ | 95/385 | 1.44 | 0.96 | - | 2.16 | 0.078 |  | 93/380 | 1.49 | 0.99 | - | 2.25 | 0.56 |

High RWTs was defined as the best-cut off value of RWTs determined by Youden index.

CI, confidence interval; GWTG, Get With The Guideline score; HR, hazard ratio; RWT, relative wall thickness.

† 2 cases were removed because of GWTG missing.
